# The Impact of Natural Disasters on Maternal Health: Hurricanes Irma and María in Puerto Rico

**DOI:** 10.3390/children9070940

**Published:** 2022-06-23

**Authors:** Irene Lafarga Previdi, Michael Welton, Jazmín Díaz Rivera, Deborah J. Watkins, Zulmarie Díaz, Héctor R. Torres, Chrystal Galán, Natacha I. Guilloty, Luis D. Agosto, José F. Cordero, Akram Alshawabkeh, Carmen M. Vélez Vega

**Affiliations:** 1 Center for Collaborative Research in Health Disparities, University of Puerto Rico-Medical Sciences Campus, San Juan 00921, Puerto Rico; carmen.velez2@upr.edu; 2 College of Public Health, University of Georgia, Athens, GA 30602, USA; mdwelton@uga.edu (M.W.); zulmarie.diaz@upr.edu (Z.D.); natacha.guilloty@upr.edu (N.I.G.); jcordero@uga.edu (J.F.C.); 3 School of Public Health, Yale University, New Haven, CT 06520, USA; jazmin.diazrivera@yale.edu; 4 School of Public Health, University of Michigan, Ann Arbor, MI 48109, USA; debjwat@umich.edu; 5 College of Engineering, Northeastern University, Boston, MA 02115, USA; hector.torres26@upr.edu (H.R.T.); chrystal.galan@upr.edu (C.G.); a.alshawabkeh@northeastern.edu (A.A.); 6 Puerto Rico Science, Technology and Research Trust, San Juan 00927, Puerto Rico; lagosto@prsciencetrust.org

**Keywords:** maternal health, emergencies, hurricanes, Puerto Rico

## Abstract

The PROTECT research Center funded by the NIH’s National Institute of Environmental Health Sciences (NIEHS) Superfund Research Program was launched in 2010 to explore the impact of exposure to pollutants on the high rate of premature births in Puerto Rico. In September 2017, Hurricanes Irma and María devastated the archipelago, which caused: collapse of the electrical system, collapse of the communication system, limited access to clean water, food, gas, and health services, destruction of public (e.g., hospitals) and private property (e.g., houses) and more than 4500 deaths. Pregnant and postpartum individuals are especially vulnerable to natural disasters. They face difficulty obtaining adequate pre- and post-natal care, are exposed to increased risk of miscarriage, premature delivery, and giving birth to low birth weight babies during and after disasters and are also more likely to suffer physical and mental health problems compared to the general population during and after disasters. A face-to-face questionnaire was administered to PROTECT participants who were pregnant during hurricanes Irma or Maria or who became pregnant shortly after in order to identify hurricane-related sources of stress and other adverse effects. This paper is based on the answers to the open-ended question at the end of the questionnaire where participants were asked to share their experiences during and after the hurricanes. Among the 375 participants who completed the survey, 76 answers to the open-ended question were considered due to data saturation. The answers to the open-ended question were transcribed into a document in order to facilitate the coding process. The transcribed text was analyzed first to identify emerging categories and then coded to identify common themes as well as divergence among participants. The following themes were identified: pregnancy and birth challenges, lack of access to basic services, housing conditions, stressful working conditions, concerns about health, concerns about their children, and positive or protective aspects. The results indicate how the disruption in access to basic services has a unique impact on the physical and mental health of pregnant and post-partum women in an emergency situation. These findings point to the potential benefit of developing specific protocols designed for emergency preparedness aimed at this population, which can inform healthcare providers and community organizations in case of future events.

## 1. Introduction

### 1.1. Maternal Health and Natural Disasters

Hurricanes can have a variety of public health implications including a surge of infectious diseases and injuries, psychosocial stress, and disruption of health care services [1] Pregnant women are considered a vulnerable population, particularly when it comes to emergency situations like natural disasters. During pregnancy, the female body goes through major cardiovascular, respiratory, gastrointestinal and hematological changes and is in constant physiological transformation [2]. In relation to maternal health, research has shown that natural disasters increase the risk of miscarriage, premature delivery, and low birth weight. In addition, pregnant individuals are more likely to suffer physical and mental health problems [3]. Studies have shown that adverse fetal outcomes increase with exposure to natural disasters [1].

Natural disasters can also cause or increase anxiety and depression among the general population due to the impact from these events such as lack of, or difficulty in access to, food, medication, and health services, as well as experiences of job loss and structural damage. According to Kirmayer and colleagues [4] there are three approaches to understanding the impact of trauma and disaster on mental health: (1) Clinical Psychiatric approach which focuses on how trauma can lead to the development of psychological conditions; (2) Individual Resources approach which focuses on the loss of resources and resilience; (3) Social Ecological approach which focuses on the role of social positioning in individual and group vulnerability. However, the psychological impact of disasters is more prevalent among vulnerable populations like children, the elderly population and women [5]. For example, studies on the impact of hurricanes have shown increased incidence of post-traumatic stress disorder and depression symptoms among pregnant women and high rates of depression among postpartum women [6,7]. In addition, there is evidence that elevated levels of depression and anxiety during pregnancy as well as psychosocial stress are associated with adverse birth outcomes [8,9].

Other vulnerabilities associated with the impact of natural disasters include the disruptions in the supply of safe drinking water, inadequate access to food, poor sanitation, and crowded housing conditions [10,11], which also impact the general population but can potentially affect pregnancy experiences and outcomes. During and immediately after disasters such as hurricanes, the focus is directed towards attending to the immediate needs of the general population, such as access to food and shelter. As a consequence, the access to healthcare services for some vulnerable populations, like pregnant or postpartum women and their newborns, can be overlooked [12].

### 1.2. Hurricanes Irma and María’s Impact in Puerto Rico

On 6 September 2017 Hurricane Irma hit the northeastern coast of Puerto Rico as a Category 5 storm, causing more than an estimated $700 million in damage, killing at least four people and causing a power outage that affected more than 1 million people [13]. Two weeks later on 20 September 2017 Hurricane María made landfall in Puerto Rico as a powerful Category 4 storm, destroying the power grid, causing more than an estimated $100 billion in damage [13]. Together, hurricanes Irma and María devastated Puerto Rico, which caused the collapse of the electrical system, the collapse of the communication system (no cellphone or internet services), and a limited access to potable water. Many people also had limited access to food and gas and other supplies and had to wait in long lines for hours in the heat to access supplies and/or rely on community support or government aid. There was limited access to health services, and there was significant damage and destruction of public and private property including hospitals and homes [14]. The toll of lives lost is estimated at over 4500 deaths [2]. These hurricanes received a limited response from the U.S. federal government as well as the local government, which made recovery more challenging [15,16].

### 1.3. Social Determinants of Health Related to Maternal Health

Social determinants such as patient factors (e.g., socioeconomic status, race and ethnicity, gender, income, behaviors, beliefs, biology, genetics), community factors (e.g., social networks and built environment, housing), healthcare provider factors (e.g., knowledge, implicit bias, communication) and systemic factors (e.g., access to high quality care, structural racism, social and political policies, healthcare institutions) play an important role in maternal health [17]. The combination of these factors serves to indicate maternal health status and can help us understand health disparities among pregnant and post-partum women. In general, social determinants of health refer to conditions in the environments in which people are born, live, learn, work, play, worship, and age that affect a wide range of health, functioning, and quality-of-life outcomes and risks [18]. These conditions affect people’s physical and mental health and their access to health-enhancing resources [19]. Emergencies such as natural disasters can affect access to medical and social services and resources. According to the Centers for Disease and Control (CDC) disasters like hurricanes or pandemics can present unique challenges to pregnant women and mothers of children 12 months or younger as well as potential exposures that can affect the developing fetus [20].

Resources that enhance quality of life can have a significant influence on population health outcomes [21]. Examples of these resources include safe and affordable housing, access to education, public safety, availability of healthy foods, local emergency/health services, and environments free of life-threatening toxins. These can be considered not only as systemic factors but also as community factors. In turn, these factors can influence patient and health provider factors. Understanding the relationship between how population groups experience “place” and the impact of “place” on health is fundamental to the social determinants of health—including both social and physical determinants [22]. When a natural disaster, like a hurricane, occurs, due to structural damage, the access to these resources is interrupted for a period of time and for pregnant and post-partum women this can result in adverse birth outcomes as previously stated. In the case of Puerto Rico, due to the slow recovery process, the lack or difficulty in accessing basic services like electricity, water and gasoline, and supplies like food, prenatal vitamins, and medications as well as adequate reproductive health services lasted for several months and impacted the pregnancy, birth and post-partum experiences of Puerto Rican women [14,23].

### 1.4. Study Objectives

1. Identify common themes among the answers of pregnant and post-partum women regarding their experiences related to hurricanes Irma and María in Puerto Rico.
2. Describe risk and protective factors experienced by pregnant and post-partum women in emergency situations.
3. Reflect on vulnerabilities related to this population and how these findings can inform future interventions and protocols for disaster preparedness.

## 2. Methods

### 2.1. Participants

The Puerto Rico PROTECT cohort is composed of pregnant women living in the North Karst Region of the Puerto Rico. The PROTECT Project is a Superfund Research Program established in 2010 that studies the role of environmental contaminants on preterm births in Puerto Rico. Women, 18 years or older, who were PROTECT participants and were pregnant between 1 September 2017 and 20 March 2018 were recruited to participate in a study of their hurricane related experiences. The study sample had a median age of 27 years (SD = 6 years); 80% indicated that they were married or living with their partner; 41% reported having a bachelor’s degree or more, and 61% reported that they were employed at the time. This was a supplemental study to the research consortium PROTECT that aimed to explore the impact of the hurricanes on pregnant and postpartum women. For the purposes of this article we used only the data from the open-ended question of the survey from a total of 375 women who were recruited for the study and we conducted a qualitative analysis.

### 2.2. Instrument

The supplemental survey on the impact of the hurricanes had questions related to continuity of services, access to healthcare, environment, hurricane experiences, and traumatic exposures. The survey also included an open-ended question at the end, which was: Is there anything else that you would like to share with us about your experience during or following Hurricanes Maria and Irma? The results presented in this paper derive from the analysis of this open-ended question.

### 2.3. Procedure

Initial contact was done through the phone by a member of the PROTECT study staff, then those women who were interested in participating were called in order to set up a meeting at their local Community Health Center to obtain an informed consent and for the administration of the survey. Answers to the open-ended question were transcribed and filed in a single document with only the participant’s ID number for identification. A total of 76 responses, from 375 participants, were considered for analysis due to data saturation which is “the point in coding when you find that no new codes occur in the data. There are mounting instances of the same codes, but no new ones” [24] or when “additional data do not lead to any new emergent themes” [24].

### 2.4. Data Analysis

The qualitative analysis consisted of a content analysis of emergent common themes across answers provided by the participants. The transcribed text was analyzed first to identify emerging categories and then coded to identify common themes as well as divergence among participants [25,26]. An initial review of the answers served to develop a codebook of emergent categories for analysis. Phrases and sentences were coded using the categories of the codebook. Two members of the research team analyzed the transcriptions and had meetings to review discrepancies and reach consensus regarding the codification of the open-ended question answers.

## 3. Findings

During the analysis process the following categories were identified in order to code the participants’ responses and understand the impact of hurricanes Irma and María and their aftermath and recovery process on maternal health in Puerto Rico.

### 3.1. Pregnancy and Birth Challenges

This category refers to the participants’ experiences related to the pregnancy process, childbirth and postpartum period. Several participants mentioned having unplanned cesareans as well as giving birth before their due date (from two weeks to two months early). One participant mentioned that her blood pressure went up during the cesarean procedure because she was worried that the power generator would stop working. This reflects the disruption of health services due to collapse of the electrical and communications systems. Being pregnant and giving birth in these conditions was very stressful for the participants; some had difficulty getting baby supplies and pregnancy related items like prenatal vitamins. One participant mentioned: “It was an agony having the birth without resources”. Another participant stated: “I was in denial of the pregnancy during the hurricane”.

### 3.2. Lack of Access to Basic Services

This category refers to the participants’ experiences related to the difficulty accessing utilities and supplies such as electricity, water, food and gasoline. Participants recalled standing for hours in order to get food supplies or gas. They also mentioned the lack of communication with their families as a source of stress. One participant mentioned that getting in touch with her husband was hard due to the lack of electricity and cellphone signal. This lack of access or difficulty in accessing basic resources was common among the general population, but certainly made participants’ pregnancy experiences more challenging. One participant commented that there were “lines for everything; water, gasoline, food. It didn’t matter that I was pregnant. Only on one occasion did they let me get ahead”. Another participant commented that “it was difficult getting gasoline in order to visit my sister at the hospital”.

### 3.3. Housing Conditions

This category refers to the participants’ experiences related to the impact of hurricanes on their homes in terms of structural damage as well as living conditions during the recovery process. Many participants lost their homes and/or belongings and had to stay with family, sometimes living with 7–9 people in small spaces with little to no privacy. One participant mentioned that she “lost everything. I stayed at his brother’s house until December. I slept on an inflatable mattress. There were seven (people) in the same house and two were babies”. The emotional impact of losing their homes or material possessions negatively impacted their well-being as well as disrupted their plans to welcome their baby in a safe home. For example, one participant mentioned that “it was horrible to spend it (the hurricane) there (at her job in generation plant) and then come home and see that I lost everything”. Another mentioned that one month after her daughter was born she got bronchiolitis and she had to move in with her mother in law to administer her treatment since she did not have electricity at her own house.

### 3.4. Working Conditions

This category refers to the participants’ experiences related to the working conditions they endured during the aftermath of the hurricanes. Several participants mentioned stressful work conditions due to lack of resources and communication, as well as exposure to contaminants. One participant, who worked as a speech pathologist, mentioned that her boss sent her out in the field, despite not having communication with the families she would attend to. Not every employer had special considerations in regards to their pregnancies. One participant expressed: “I was exposed to the fungus and everything contaminated in the store”. Another one mentioned that she worked at a hospital but the patients were not arriving, yet she was still asked to come in; the structure had suffered damages such as broken doors and windows. At other times, their partners’ working conditions were mentioned as a source of stress due to the lack of communication and general uncertainty during the immediate aftermath.

### 3.5. Concerns about Health

This category refers to the participants’ experiences related to concerns about their own health as well as the health of their partners, family and children. Most participants reported feeling stressed during the recovery from the hurricanes. One participant said that “I was traumatized”, another said “I fell into a horrible depression”. Meanwhile another participant expressed that “I am scared every time I see Ada Monzón (local meteorologist)”. This demonstrates the negative impact of the hurricane aftermath on their mental health which makes recovery more challenging and could have long term impacts. Some got sick from drinking contaminated water and others lost family members due to lack of access to proper healthcare. One participant mentioned that she was “hospitalized for diarrhea and vomiting. Before that I drank spring water, then I switched to bottled”. The lack of access to basic resources put them and their loved ones at risk of illness, injury and in some cases even death. One participant mentioned that her grandmother died on 27 September because “she couldn’t get her oxygen tank (working). It was a respiratory arrest”.

### 3.6. Concerns about Their Children

This category refers to the participants’ concerns regarding the impact of the hurricanes on their children’s overall wellbeing. Several participants expressed concerns about their children’s behaviors and moods. One participant recalled that “trying to stay calm was difficult; I felt compelled to do so for my son who had just turned 4 years old. I consider that my son has changed his mood because of all this”. This participant reported that she had to take her child to see a psychologist and is presently in treatment. Meanwhile another participant mentioned that when they returned from staying in Florida, her daughter “was left without a teacher at her school”. She considers that after transferring her to another school, her attitude changed. Others stated that their children are presently more attached to them than before the hurricanes. One participant mentioned that “they tried to put her in child care, but it didn’t work. She is too attached”. These expressions reflect the impact of these experiences on young children, who while not being old enough to fully comprehend the severity of the situation can sense the disruption of routines and thus fear for their safety.

### 3.7. Protective Factors

This category refers to the participants’ experiences related to interpersonal relationships with their family and neighbors, the material and emotional support during recovery, as well as the aid received from local and/or federal government. These protective factors helped them access basic resources like food, supplies, electric generators and even housing and thus ensured basic needs were met. For example, one participant mentioned that the municipality gave them supplies like milk and diapers; another one mentioned that FEMA gave them $300 to fix their door. Another participant mentioned that her husband’s job provided them with a generator, food, water and ice. Many participants expressed that the support from family and neighbors helped them considerably. One mentioned that their neighbors were very attentive, they would ask if they had eaten and also give them food. Another mentioned that the PROTECT Project had given them a mosquito repellent and a net. Others mentioned that the lack of resources led to a sense of community and bonding activities. One participant remembered: “We played cards and board games. At the same time, it was good because we spent time as a family”. Another stated “the only thing I miss about the post-Maria period is seeing how people got together more. They got together to play STOP and the children played outside. When the electricity arrived, all that ended”.

## 4. Discussion

The results indicate how the disruption in access to basic services may impact the physical and mental health of pregnant and post-partum individuals in an emergency situation. The participants mostly expressed how working and housing conditions and lack of access to basic services like water, electricity, and Wi-Fi during the aftermath of Hurricanes Irma and María influenced concerns about their wellbeing and that of their families, and affected their birth and post-partum experiences. Puerto Rico was in a vulnerable position due to the current economic crisis, a deficient infrastructure, local corruption and its political status as a U.S. colony [27]. Thus, it was not adequately prepared for hurricanes Irma and María [28]. Slow and inefficient recovery response exacerbated the negative impact on the physical and mental health of participants. However, the participants’ discussion of protective factors such as the interpersonal relationships with their family and neighbors and the material and emotional support they received from them during recovery reflect the resilience of the people of Puerto Rico and the importance of community and solidarity in recovery efforts [29].

As expressed earlier in the article, hurricanes and tropical storms may have lasting implications, including economic crises, psychosocial stress, and disruption of health care services as well as of basic services like electricity and water [1]. Pregnant and post-partum women are more vulnerable in comparison to the general population due to the physiological and psychological changes that occur during this period and the need for continuous perinatal care [5]. Several study participants mentioned how the hurricanes’ aftermath affected their birth plans and how stressful it was to give birth and have a new born baby without access to basic resources. Some participants described the negative impact of the hurricanes on their mental health as well as the difficulty in getting supplies such as prenatal vitamins. Other participants mentioned that they did not receive any special treatment at work and some had to wait for supplies in spite of being pregnant. In general, during the recovery phase after disasters such as hurricanes, the focus is directed towards addressing the immediate needs of the general population, such as food and shelter. As a result, the access to healthcare services and other resources for pregnant or postpartum women and their newborns can be overlooked [12].

These findings point to the relevance of social determinants of health when considering the needs of vulnerable populations during emergencies and recovery situations. Social determinants such as patient factors (e.g., pregnant women between 18–40 years living in the Northern Karst region of Puerto Rico), community factors (e.g., social support from family and neighbors, workplace), healthcare provider factors (e.g., lack of access and communications due to hurricanes) and systemic factors (e.g., economic crisis, deficient infrastructure, slow and inefficient recovery) influenced the participants’ experiences of the effects of the hurricanes [17]. Thus, policymakers should take into consideration these social determinants of health when designing protocols and policies for disaster preparedness.

According to the literature there is a need for improvement and implementation evidence-based interventions related to disaster preparedness to address the needs of pregnant women and mothers of young children [30,31]. Protective factors should be considered in preparation for emergency situations since they can help minimize adverse effects. For example, social support is considered a relevant protective factor that can positively impact the recovery process [6,32]. According to Kirkmayer and colleagues [4], depending on the degree to which a disaster disrupts normalcy, communities may respond with mobilization and increased solidarity or with demoralization and disorganization. In the case of Puerto Rico, as evidenced in the participants’ responses as well as news articles and research studies, what happened was the former. In the face of a slow and inefficient response from institutions, it was the people who helped each other. Thus, recovery efforts should help build social support networks within the communities affected by the hurricanes. Enhancing social support in these communities is likely to have a positive impact on the overall wellbeing of pregnant and postpartum women and their families.

Access to resources is another protective factor that is important to consider. Some lessons learned from the study participants’ experiences of the hurricanes were the importance of planning ahead and identifying possible needs and existing resources. Some participants mentioned that they were not adequately prepared for the hurricanes and as a result learned the importance of emergency preparedness instead of just recovery efforts. Thus, individual, community and state responsibilities in terms of access to resources need be addressed during emergency preparedness. For example, in PROTECT we have provided participants, after consulting them, with materials like water filters, mosquito nets, powdered milk, and baby products, as well as educational material related to emergency preparedness aimed at pregnant and post-partum women (i.e., infographics from CDC and local authorities, materials developed by the PROTECT team) [33]. In addition, state and local agencies and institutions must work on emergency preparedness that responds to the specific needs of diverse communities and populations such as pregnant and post-partum women and provides them with access to resources [18,34]. This issue needs to be addressed since little progress has been made on this front since the hurricanes hit Puerto Rico in 2017 [35]. In summary, individuals, communities and government have different roles during an emergency in terms of providing support and access to resources and this has to be taken into consideration in emergency response and in research that develops and evaluates evidence-based interventions.

Finally, in terms of the study limitations, we acknowledge that the sample did not include participants from all areas of the archipelago, but only focused on women from a specific region in Puerto Rico. This was also a qualitative study with a small sample size, which precludes causal inferences. Another limitation is the recall of events and experiences, since the questionnaire was administered after the hurricanes had already passed. In addition, some themes were not explored in the open question since they were covered in the questionnaire questions. These topics included access to healthcare services, environmental exposure and traumatic experiences, and will be reported in other publications. In conclusion, study findings point to the potential benefit of developing specific protocols designed for emergency preparedness aimed at pregnant and postpartum women, which can inform healthcare providers and community organizations in the case of future events. These protocols should be culturally relevant and appropriate, while taking into consideration the socio-economic and political context. Finally, it is important to take into account social determinants of health in disaster preparedness and response, while promoting changes at the individual, community, and system levels.

## Data Availability

The datasets used and/or analyzed during the current study are available from the corresponding author on reasonable request.
